# Autoinfarction of Giant Parathyroid Adenoma after Preoperative Withdrawal of Anticoagulants

**DOI:** 10.1155/2018/9261749

**Published:** 2018-10-23

**Authors:** Raoul Verzijl, Pim J. Bongers, Geetha Mukerji, Ozgur Mete, Karen M. Devon, Jesse D. Pasternak

**Affiliations:** ^1^Division of General Surgery, Department of Surgery, University Health Network, Canada; ^2^Division of Endocrinology, Department of Medicine, Women's College Hospital, Canada; ^3^Department of Pathology, University Health Network, Canada; ^4^Department of Surgery, Women's College Hospital, Canada

## Abstract

A 71-year-old man with known history of atrial fibrillation (treated with routine rivaroxaban therapy) was found to have incidental biochemical elevated calcium and parathyroid hormone (PTH) levels. His physical examination demonstrated the presence of a palpable right neck mass. Subsequent imaging studies revealed a large parathyroid mass as well as multiple bone lesions, raising the suspicion of parathyroid carcinoma. The anticoagulant therapy was stopped 5 days prior to his elective surgery. The night before his elective surgery, he presented to the emergency room with profound hypocalcemia. The surgery was postponed and rescheduled after calcium correction. Intraoperative findings and detailed histopathological examination revealed an infarcted 4.0 cm parathyroid adenoma with cystic change. His bony changes were related to brown tumors associated with long-standing hyperparathyroidism. Autoinfarction of a large parathyroid adenoma causing severe hypocalcemia is a rare phenomenon and may be considered in patients with large parathyroid adenomas after withdrawal of anticoagulants.

## 1. Introduction

Primary hyperparathyroidism (pHPT) is the most common cause of hypercalcemia in the outpatient, with an incidence between 27 and 30 per 100,000 person-years [1]. In over 80% of patients, a single benign parathyroid proliferation (parathyroid adenoma) causes autonomous hypersecretion of parathyroid hormone (PTH). This leads to an elevated serum calcium concentration with concordant manifestations such as renal stones and osteoporosis. Patients rarely present with classically advanced symptoms of hyperparathyroidism such as osteitis fibrosa cystica and lytic lesions, called brown tumors. Although common before high-throughput calcium testing became available in the 1970s, these bone lesions are rarely seen today and can confuse the clinical picture by suggesting metastatic disease.

In addition to illustrating this diagnostic uncertainty, this case report describes a scenario where severe hypercalcemia due to primary hyperparathyroidism was reversed following tumor autoinfarction after cessation of oral anticoagulation.

## 2. Case Report

A 71-year-old man, originating from Vietnam, was referred to a tertiary care endocrine surgery center with incidentally found severe hypercalcemia of 3.65 (reference range, 2.20–2.62) mmol/L during routine follow-up for atrial fibrillation. His only symptoms of hypercalcemia on further questioning were fatigue and polyuria. The patient's history included the left leg dystrophy due to childhood polio, hypertension, atrial fibrillation, chronic kidney failure (stage IIIB), and dyslipidemia. He had no family history of parathyroid or other endocrine disease, and he had no prior neck irradiation.

**Figure 1:**
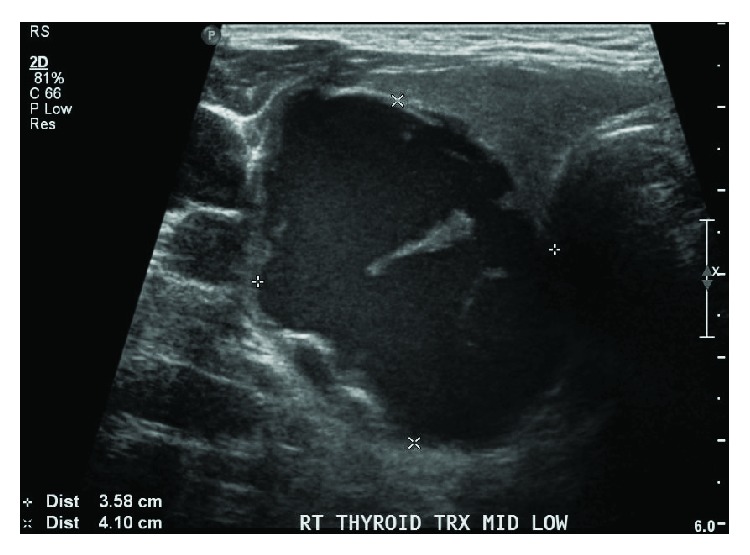
Ultrasound showing the parathyroid mass.

On physical examination, a palpable mass was noted on the right lower neck without associated palpable lymphadenopathy. Flexible nasolaryngoscopy showed normal vocal cord movement, suggesting no invasion into the recurrent laryngeal nerve. Laboratory results showed markedly elevated levels of calcium at 3.3 mmol/L, PTH at 271 (1.4–7.6) pmol/L, alkaline phosphatase (ALP) at 446 (40–150) U/L, and phosphate of 1.06 (0.8–1.4) mmol/L. Ultrasound of the neck showed a complex, predominantly cystic nodule with solid components inferior of the right thyroid lobe measuring 4.1 × 3.6 × 3.1 cm (Figure 1).

**Figure 2:**
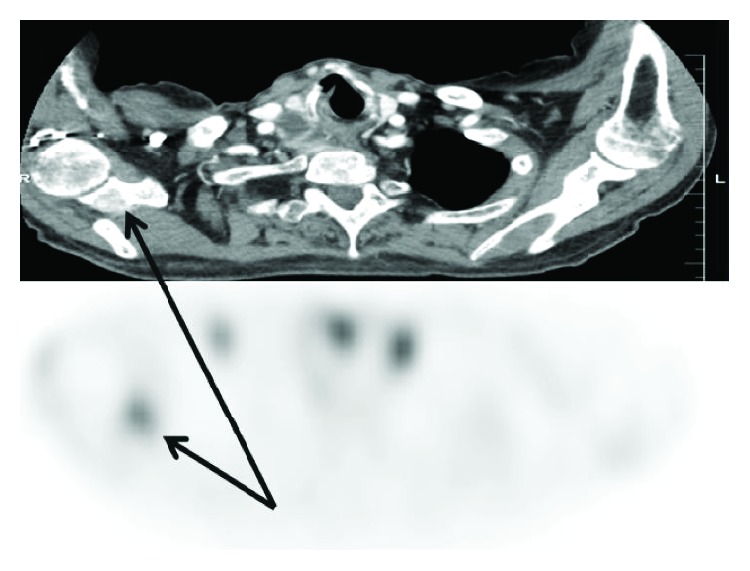
CT scan (a) shows one of the bone lesions that has increased tracer uptake on the (99m)Tc-sestamibi scan (b).

Sestamibi scan ((99m)Tc) displayed a dominant right-sided neck lesion with minimal uptake of technetium. Additionally, two well-defined lucent bone lesions, located in the right clavicle and scapula, showed increased tracer uptake (Figure 2). Subsequent CT scans of the neck, thorax, and abdomen and a bone scintigraphy confirmed the presence of two previously defined osseous lesions along with two additional lucent and sclerotic lesions in the sternum and 7^th^ rib. Differential diagnosis of the bone lesions included both metastasis as well as brown tumors secondary to long-standing hypercalcemia related to hyperparathyroidism. We did not evaluate the lesion with FNA preoperatively.

**Figure 3:**
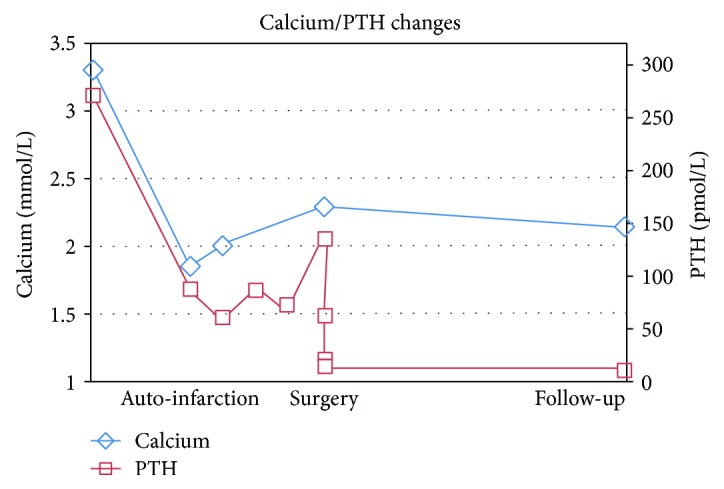
Course of calcium and PTH levels related to the different events.

Due to the clinical suspicion of parathyroid cancer, en bloc parathyroidectomy with the right thyroid lobectomy and ipsilateral level VI lymphadenectomy was planned. Rivaroxaban, a direct Xa inhibiting anticoagulant indicated for atrial fibrillation, was stopped 5 days prior to surgery. The evening before the scheduled surgery, the patient presented to the emergency department with symptomatic hypocalcemia including perioral, finger, and toe numbness. Laboratory results showed profound hypocalcemia with a corrected calcium level of 1.85 (2.32–2.62) mmol/L, phosphate of 0.61 (0.8–1.4) mmol/L, and a decrease in PTH level to 87.5 (1.4–7.6) pmol/L (Figure 3).

Repeated ultrasound showed further avascular solid components of this parathyroid mass but no changes in size of the lesion. Parathyroid tumor autoinfarction was suspected with concomitant hungry bone syndrome. The surgery was postponed, and the patient was treated with high-dose calcium supplementation and activated vitamin D. After eucalcemia was achieved, the originally planned surgical intervention was performed.

Rapid intraoperative PTH assays showed a significant drop of PTH after the removal of this mass (baseline intraoperative PTH, 134 pmol/L; 10-minute sample, 13.8 pmol/L). During the surgery, we used intraoperative nerve monitoring (IONM) for assessment of the right recurrent laryngeal nerve. The nerve signal was not lost throughout the operative dissection, and there were no adhesions to the recurrent laryngeal nerve from the parathyroid lesion. Postoperative movement of vocal cords was normal, calcium levels remained stable, and the patient was discharged 1 day after surgery.

**Figure 4:**
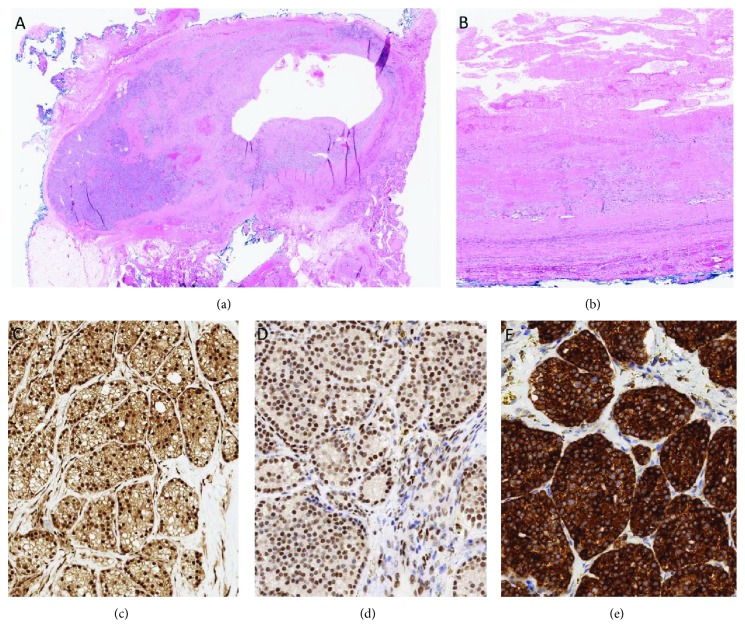
The specimen showed a largely infarcted enlarged cellular parathyroid gland with cystic change (a, b; hematoxylin and eosin). There was no evidence of invasive growth. There was no loss of expression for parafibromin (c), RB (d), p27 (not shown), and bcl-2 (e). The overall clinicopathological findings were consistent with an infarcted parathyroid adenoma.

The right hemithyroidectomy specimen identified a 4.0 cm mass located at the right perithyroidal region. The mass was an infarcted enlarged cellular parathyroid gland with cystic degeneration (Figure 4). There was no evidence of angioinvasion or malignant invasive growth to suggest the morphological diagnosis of parathyroid carcinoma. Given the clinical suspicion for malignancy and underlying infarction, this lesion was further assessed with immunohistochemical biomarkers (Figure 4) [2–5]. The lesion was positive for PTH (confirming the parathyroid origin) and was negative for galectin-3 and PGP9.5. There was no loss of expression for parafibromin, Rb, p27, and bcl-2. The MIB-1 labeling index was 1%. The mitotic activity was 0.6 per 10 high-power fields (based on 3 per 50 high-power fields). The immunoprofile also did not suggest malignancy. While no atrophic rim of parathyroid tissue was identified, submitted additional parathyroid tissue from the right neck and significant drop of intraoperative PTH were suggestive of a single gland disease, consistent with an infarcted parathyroid adenoma [6].

At the 6-month follow-up, calcium and PTH levels remained improved and no persistence or recurrence of disease was suspected.

## 3. Discussion

Hypocalcemia after autoinfarction of a parathyroid adenoma is an extremely rare entity [7]. This is the first report, to our knowledge, of discontinuation of anticoagulants causing autoinfarction of a large parathyroid tumor and resulting hungry bone syndrome.

Previous literature proposed that fragile vascularization of parathyroid adenomas can lead to subsequent autoinfarction. The mechanism of this occurring in large parathyroid adenomas is focused on the solid aspects of parathyroid tissue, which are most sensitive to changes in vascular supply. This occurs around the rim of the tumor, while the center usually undergoes cystic degeneration [8]. The ultrasound done in this patient was consistent with this proposed explanation [9].

Although calcium levels can normalize after autoinfarction of a parathyroid adenoma, surgical intervention is still warranted. This is based on previous studies where calcium normalization after autoparathyroidectomy with subsequent nonsurgical surveillance showed recurrence of hyperparathyroidism [10, 11]. A short delay of surgery is suggested as inflammation and scar tissue around parathyroid tumors could increase the surgical risk of injury to adjacent structures such as the trachea or recurrent laryngeal nerve [12]. Further, eucalcemia should be obtained before further subjecting the patient to changes in calcium homeostasis with adenoma resection.

The physiologic process of hypocalcemia after autoparathyroidectomy can be explained by the shift in balance of bone metabolism from osteoclastic bone resorption to enhanced osteoblastic bone formation due to the sudden relative decrease in PTH levels. Enhanced bone formation requires increased calcium uptake and consequent lower levels of circulating calcium. Patients with previous manifestations of high bone turnover or bone disease are prone to develop hungry bone syndrome after parathyroid resection, specifically those with high baseline ALP levels preoperatively as in this patient [13].

One issue of diagnostic uncertainty was the presence of bone lesions signifying possible metastatic disease which was later determined to be brown tumors. These benign lesions, seen in up to 3% of pHPT patients, represent a reparative cellular process in regions with high osteoclastic bone turnover [14]. Rapid healing of these lesions is common once the underlying hyperparathyroidism is corrected.

Finally, the lack of reliable tools to determine preoperative malignancy risk makes management of large parathyroid tumors difficult. Although parathyroid malignancy is the cause of pHPT in only 1% of the patients, it must be considered when levels of calcium and PTH are highly elevated in combination with a large palpable neck mass, especially if there is clear evidence of locoregional invasion or distant metastasis. Unfortunately, fine needle aspiration (FNA) cytology is unsuitable for the evaluation when parathyroid carcinoma is suspected. FNA can lead to tumor seeding and potentially cause parathyromatosis. In addition, previous interventions including FNA can also result in worrisome histological changes simulating malignancy [15]. Therefore, careful intraoperative surgical evaluation and en bloc resection followed with a thorough histopathological examination are essential if there is suspicion of parathyroid malignancy. Operative plan usually includes resection of the parathyroid mass with the ipsilateral thyroid lobe and ipsilateral level VI lymph nodes. If there is clearly no invasion into the adjacent structures, limited resection has been advocated specifically without an ipsilateral thyroidectomy.

Pathological analysis integrating the use of ancillary biomarkers remains the best diagnostic tool for malignancy [2–4].

This case report

- reviews the clinicopathological work-up and operative approach to primary hyperparathyroidism when there is suspicion of carcinoma
- shows that autoinfarction of parathyroid tumors can mimic operative ablation in terms of physiologic effects on calcium homeostasis and that this should be considered in patients with significant decrease in calcium levels preoperatively, especially after withdrawal of anticoagulants
- emphasizes that bone can be profoundly involved in benign parathyroid disease in the form of brown tumors, signifying long-standing hyperparathyroidism
- highlights the clinical challenge in distinguishing benign and malignant parathyroid tumors due to the lack of definitive preoperative diagnostic tools
